# Conservative treatment of a scoliosis patient after two heart surgeries in early childhood – A case report

**DOI:** 10.4102/sajp.v77i2.1588

**Published:** 2021-11-30

**Authors:** Hans-Rudolf Weiss, Manuel Lay, Tamisha Best-Gittens, Marc Moramarco, Mario Jimeranez

**Affiliations:** 1 Schroth Best Practice Academy, Neu-Bamberg, Germany; 2 Koob Scolitech GmbH, Neu-Bamberg, Germany; 3 Orthopedic Technology, Orthopädietechnik Lay GmbH, Zell-Barl, Germany; 4 ScolioPhysio Caribbean, Christ Church, Barbados; 5 Scoliosis3DC/Private Practice, Woburn, United States of America; 6 Private, Sevillano, Havanna, Cuba

**Keywords:** congenital heart disease, scoliosis, physiotherapy, brace treatment, cosmesis

## Abstract

**Introduction:**

This is a case report of a juvenile female patient with scoliosis following two heart surgeries for congenital heart disease (CHD).

**Patient presentation, management and outcome:**

Initially, the premenarchial female was 9 years old and had a Tanner stage 2–3 with a single thoracic curve of 65° Cobb. Because of the high risk for progression, immediate brace treatment was proposed as the father declined surgery. The patient received intensive treatment according to the Schroth Best Practice® programme and a Gensingen Brace® designed for large thoracic curves. Over the 18 months following the initial visit, she received two additional braces. As a result, the progression of the main curve was prevented. The patient continues to maintain an improved cosmetic result and is currently at a Risser 2.

**Conclusion:**

Surgery performed for CHD in rare cases may lead to stiff spinal deformity as a consequence of that surgery. Progression of a severe and stiff curve was prevented during the most vulnerable phase of the pubertal growth spurt with an improved clinical result. Therefore, we assume that the patient may have a normal life in adulthood with minor restrictions only. Supported by pattern-specific high correction exercises and braces, these typical single thoracic curves can be re-compensated to a more balanced appearance, less prone to progression in adulthood.

**Clinical implications:**

Because of the relative high risks of spinal fusion and the long-term unknowns of such an intervention, high-impact conservative treatment should be implemented first before surgical correction is considered.

## Introduction

The term scoliosis describes a three-dimensional deformity of the trunk and spine. In phases of high-growth velocity, it may deteriorate dramatically (Asher & Burton 2006; Goldberg et al. 2002). A variety of causes may lead to symptomatic scoliosis, for example, congenital scoliosis, neuromuscular scoliosis, scoliosis in mesenchymal disorders and many other underlying diseases or syndromes (Chik 2020). However, 80% – 90% of all scoliosis cases are denoted as idiopathic scoliosis and are of unknown origin (Asher & Burton 2006; Goldberg et al. 2002).

Treatment for scoliosis consists of physiotherapy, brace treatment and surgery. There are some studies supporting pattern-specific exercises (Kuru et al. 2016; Monticone et al. 2014); however, the treatment effects are low, and there remains no evidence that physiotherapy may alter the course of scoliosis greater than 20° during the high-risk phase of the pubertal growth spurt.

Brace treatment is supported by a randomised study (Weinstein et al. 2013) and by prospective controlled investigations (Nachemson & Peterson 1995; Weiss & Weiss 2005). It should be noted that different brace designs lead to different outcomes (Weiss & Turnbull 2020). Reliable computer-aided design or computer-aided manufacturing (CAD or CAM) can be used to standardise brace treatment (Weiss et al. 2021).

Scoliosis may also be a consequence of heart surgery in early childhood for congenital heart disease (CHD). According to Ogilvie (1995), CHD occurs in approximately 7 per 1000 live births, and the incidence of scoliosis amongst individuals surviving into childhood has been reported to be as high as 12% if all curves greater than 10° are considered. Individuals with scoliosis greater than 20° account for less than 5% of the patient population and in patients with curves exceeding 25° and residual growth, brace treatment is indicated like in patients with adolescent idiopathic scoliosis (AIS) (Ogilvie 1995).

According to recent publications, scoliosis following heart surgery is variable (Feiz et al. 2012; Kaito et al. 2018). There are contradictory publications regarding the incidence of scoliosis in patients with CHD after heart surgery (Feiz et al. 2012; Kaito et al. 2018).

In patients with CHD, the thoracotomy causes scoliosis, and there is a high prevalence of scoliosis in patients with CHD surgically treated through a median sternotomy (Ruiz-Iban et al. 2005). The prevalence of scoliosis increases in patients operated at an early age (Ruiz-Iban et al. 2005). Tsirikos et al. (2020) state that there is limited information on spinal care for patients with CHD.

The purpose of our article is to present a case with CHD and a collapsing spine treated conservatively at the start of puberty.

## Case presentation

The first author was contacted by the father of the patient who presented pictures of his daughter with scoliosis appearing after two heart surgeries for CHD, one at the age of 5 months and the other at 12 months. According to the patient’s father, the patient’s cardiopulmonary performance was in the normal range after the second heart operation and has remained normal to this day. Except for the progressive scoliosis, there were no other noticeable findings.

**FIGURE 1:**
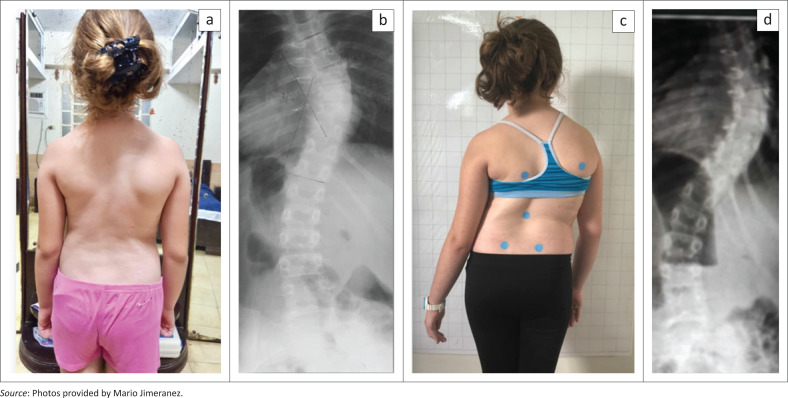
Significant progression of the curve within 18 months from 45° (a and b) to approximately 65° Cobb (c and d). Whilst the trunk of the patient was compensated at the age of eight (a), at the start of treatment at 9.9 years, the patient showed signs of a collapsing spine with a drastic decompensation of the trunk to the right side.

At the time of the first e-mail contact with the first author, the premenarchial female was 9 years old (date of birth: 02 February 2010) and had a Tanner stage 2–3 with a single thoracic curve of 65° Cobb. Unfortunately, the upper thoracic spine was not visible on the initial X-ray. Therefore, the Cobb angle measurement may have been prone to a technical error on this X-ray. Compared to the X-ray taken in April 2018 when the patient was 8 years old, the main thoracic curve had progressed by 20° Cobb (Figure 1).

Because of the immediate risk for further progression, brace treatment was initiated immediately as proposed by the first author because the father had declined surgical intervention as suggested by a spine surgeon in Cuba.

The patient was able to travel to Barbados (November 2019) to receive intensive treatment according to the Schroth Best Practice™ programme as well as a special Gensingen brace™ designed for large thoracic curves, both given by the third author. A photo documentation was created prospectively from the start of treatment to the last check-up appointment, and the Cobb angle was documented, as was the angle of trunk rotation (ATR) according to Bunnell (2005). No pain was reported at the time of admission. As there were no neuromuscular dysfunctions and because of the time limit for the father and daughter’s stay, extensive muscle and functional tests were dispensed with. Quality of life questionnaires was also not used in this 9-year-old patient.

The third author found free mobility of the extremities as well as of the cervical and lower lumbar spine and a very significant impairment of movement in the thoracic area. The entire thoracic spine could only be slightly straightened in a manual flexion test. The right-side flexion was thus drastically restricted, the left-side flexion only to a minor extent. Manual derotation of the rib hump was hardly possible.

The patient’s torso was scanned with a hand scanner for the brace fitting, and the measurement data required for the fitting (ap, ll and length measurements between specific torso regions) were also determined. These were immediately forwarded to initiate the brace supply.

Then father and daughter were instructed in the Schroth Best Practice™ Programme for independent further treatment at home. The treatment was 90 min per day and was carried out for 5 days.

The brace was manufactured in the USA under the supervision of the fourth author and arrived in Barbados within 4 days. The brace was adjusted by the third author, and the fine adjustment was made with the help of the first author via Skype.

**FIGURE 2:**
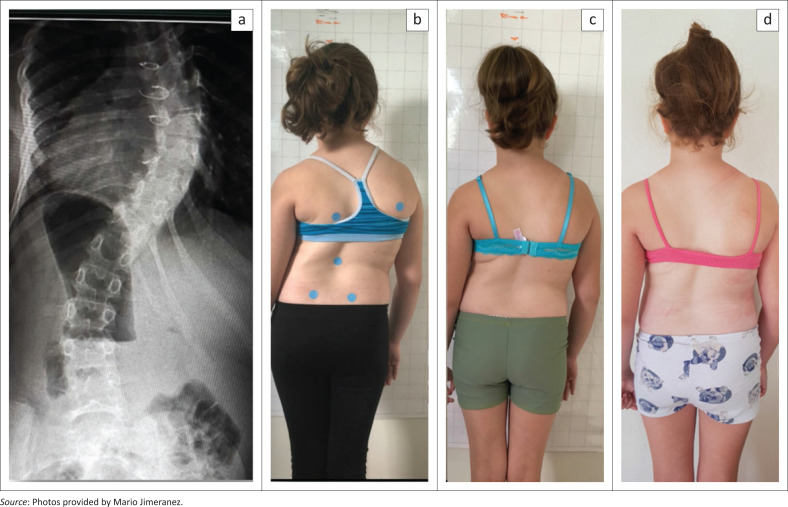
The patient with two operations for congenital heart disease with a collapsing spine at the age of 9.9 years at the start of treatment. (a) X-ray taken on November 2019 with a Cobb angle of approximately 65° Cob, (b) picture from the same time showing the patient from the rear with a collapsing spine, (c) after the application of the Schroth Best Practice Programme whilst waiting for the brace and (d) after a few nights spent in the brace.

The in-brace correction was moderate (50° Cobb) as expected because of the underlying condition, but within a short period, a reasonable clinical improvement had been achieved initially with the help of pattern-specific physiotherapy (Schroth Best Practice programme) whilst waiting for the brace to arrive in Barbados (Figure 2).

**FIGURE 3:**
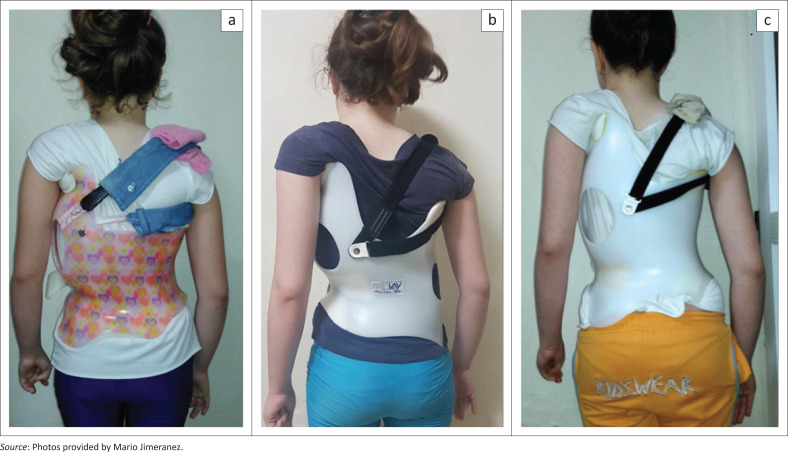
The three braces made for the patient. (a) A Gensingen brace pattern 3C for large curves (11/2019), (b) a Gensingen brace pattern 4C to address the clinical pattern change (9/2020) and (c) third Gensingen brace pattern 3C again (2/2021).

Over the following 18 months, the patient received two additional braces sent to Cuba from Germany, both manufactured by the second author (Figure 3). Because of the appearance of a lumbar counter curve, the second brace was made for a double major pattern of curvature (August 2020) as the patient – still at Risser 0 – was at high risk for a progression of this secondary lumbar curve. Prior to the third brace (February 2021), another X-ray had been taken revealing a Cobb angle of 67°. The brace adjustments were made by the latter author and were monitored by the first author via Skype. Changes to the findings were also discussed with the first author via Skype, and corresponding changes to the brace were regularly initiated.

The patient was instructed to initially perform the corrective exercises learned twice a week for 30 min at home to support the brace correction and to mobilise the thoracic spine. The exercises were performed under the supervision of the father, ensuring compliance.

Since June 2021, the patient has been able to reduce the brace wearing time to 16 h per day because of the reduced growth rate and the now more favourable prognosis. From this point on, daily exercises were prescribed considering the learned corrective everyday postures (ADLs).

### The physiotherapy programme applied

The physiotherapy programme used with this patient is a further development of the Schroth method (Kuru et al. 2016). The so-called Schroth Best Practice™ (SBP) programme (Weiss et al. 2018) contains several modules that merge into an individual, pattern-specific overall concept. The aim of the treatment is to enable the patient to carry out the exercise programme independently at home without too many aids. The training of everyday activities is of particular importance. The treatment modules are the following:

- physio-logic exercises
- pattern-specific everyday training (ADL)
- pattern-specific so-called ‘3-D made easy’ movements (in preparation of the next module)
- pattern-specific three-dimensional correction exercises (Schroth/Schroth Best Practice)
- transfer of ADLs in locomotion
- so-called de-tethering exercises to promote the mobility of the nervous system.

The Schroth method today is used worldwide. It has been extensively investigated and can be considered as being evidence based (Kuru et al. 2016; Weiss et al. 2018).

### The brace applied

The Gensingen Brace® is of CAD with a library of different brace modules for different curve patterns (Weiss & Kleban 2015). The brace library is based on the augmented Lehnert–Schroth (ALS) classification (Weiss et al. 2019). Following virtual adjustment of the braces to the patient’s trunk scan, the virtual braces are individualised with respect to correction by carefully trained brace designers. The designs are provided by Koob Scolitech GmbH, Germany, for specialised bracing centres around the globe.

After verification of the brace measurements in comparison to the patient’s measurements on a computer, the STL-file of the brace is extracted. This file can then be read into a computer numerical control (CNC) milling machine. The brace shape is then milled onto a polyurethane foam body that serves as the starting point for the production process. This foam body is used for vacuuming (thermoforming) the final brace, which can be adjusted to the patient’s body after being cut from the foam model with adequate finishing steps. The individual steps for producing CAD or CAM braces can be found in Weiss et al. (2017b).

A PubMed review was carried out to obtain an overview of the existing evidence on the treatment of scoliosis in patients with CHD. The search string ‘scoliosis, congenital heart disease’ displayed 610 results. However, the results were very unspecific. Almost a third of the results related to Marfan’s syndrome that has nothing to do with our case presentation. It turned out that the specific literature on CHD can only be found by using the commonly used abbreviation CHD.

The search string ‘scoliosis, CHD’ resulted in 25 citations, and the search string ‘scoliosis, CHD, treatment’ produced 15 citations, but only eight citations for the treatment of scoliosis in patients with CHD after heart surgery. These citations only highlighted the results and complications of surgical interventions. Neither the search string ‘scoliosis, CHD, physiotherapy’ nor the search string ‘scoliosis, CHD, brace treatment’ revealed a result.

### Outcomes

Progression of the main curve was prevented during the most vulnerable phase of growth (no change within the margin of ±5°). The patient continued an improved cosmetic result prior to the third brace at Risser 2 (Figure 3). The brace was designed for a single thoracic curve like the first brace to attempt re-compensation of the upper trunk.

**FIGURE 4:**
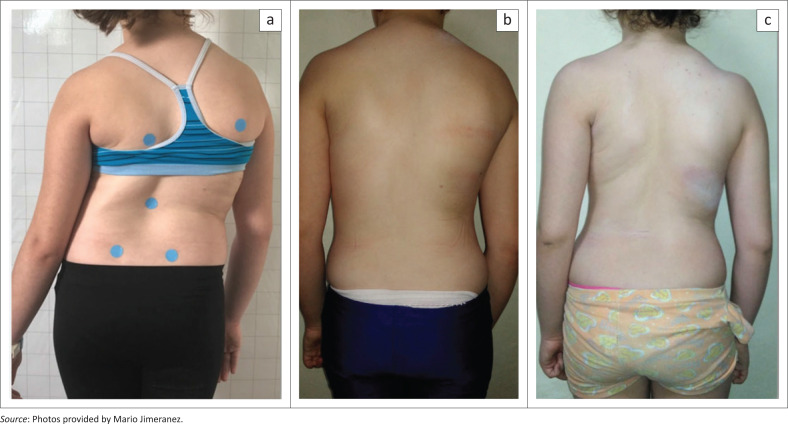
Clinical aspects of the patient during the follow-up. (a) Initial finding (November 2019), (b) intermediate result with pelvic prominence to the right indicating a clinical 4C pattern and (c) clinical result of the patient at Risser 2 prior to application of the third brace (2/2021).

The ATR as measured with a scoliometer (Bunnell 2005) after the first improvement during the first 6 weeks of conservative treatment did not change to a wide extent (Table 1). The clinical changes are documented in Figure 4.

**TABLE 1:** Variation of the angle of trunk rotation (ATR) as measured with the Scoliometer®.

Value at the start	22
At 6 weeks	15
At 12 weeks	14
At 18 weeks	15
At 24 weeks	15
At 30 weeks	15
At 36 weeks	16
At 42 weeks	15
At 48 weeks	15
At 54 weeks	15
At 60 weeks	14

Source: Table provided by Mario Jimeranez.

## Discussion

Our case study appears to be the first published on conservative treatment in a patient with stiff spinal deformity because of surgery for CHD. We did not find any paper related to this topic in PubMed.

Few papers have been published with respect to surgical correction for patients with scoliosis following heart surgeries for CHD. In some studies, a high rate of complications has been documented (Cohen et al. 2020; Pérez-Caballero 2009; Przybylski et al. 2019) and this patient population is also at risk for impaired psychosocial functioning because of repetitive operative procedures (Hu et al. 2020). Low complication rates were found by Spitzer et al. (2019). However, no long-term study of this patient population after scoliosis correction was found, and there is no evidence that patients may benefit from scoliosis surgery in general (Bettany-Saltikov 2015; Cheuk et al. 2015; Hawes 2006; Ward et al. 2017). However, there is some evidence that the adverse long-term consequences of scoliosis surgery exceed the adverse long-term consequences of the deformity (Hawes 2006; Cui et al. 2012; Lau et al. 2014).

For patients with an operative scoliosis correction after having undergone heart surgery for a CHD, no long-term outcome studies exist. Therefore, it is reasonable to implement high-quality conservative treatment first before surgery is considered. There is some evidence that patients with idiopathic scoliosis can be treated successfully even in curves exceeding 40° (Aulisa et al. 2019; Weiss et al. 2017a, 2021). The question arises as to whether patients with stiff spinal deformities because of surgery for CHD may also benefit from non-operative interventions.

According to Goldberg et al. (2002), two-thirds of the progression of a scoliotic curve happen in the ascending phase of the pubertal growth spurt from the onset of the first signs of maturation (Risser 0; Tanner 2 to Tanner 3). One-third of the progression occurs in the descendent phase of the pubertal growth spurt (Tanner 3–5 or Risser 1–5). This means that the prognosis for the patient described herein now at Risser 2 is better than at the start of treatment (Tanner 3 or Risser 0).

The ATR as measured with the Scoliometer® (ATR) as an objective measure for cosmesis (Bunnell 2005) improved significantly in a recent study for patients with an AIS and curves exceeding 40° (Weiss et al. 2021). This indicates that the cosmetically important parameters usually can be improved with high correction braces. However, this value only describes the trunk asymmetry when the trunk is flexed forward. Even if this value does change to a considerable extent, the improvement in trunk asymmetry in the upright position is clearly visible in this case (Figure 4).

**FIGURE 5:**
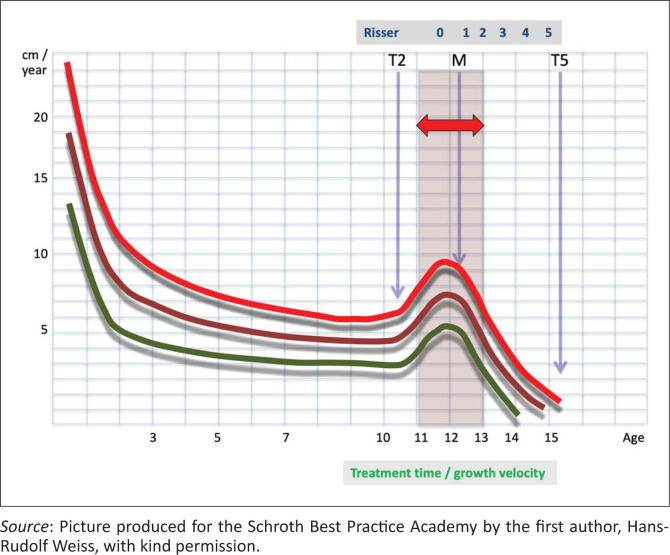
Follow-up during the treatment period for the 9.9-year-old premature patient with Tanner 2–3 at the start of treatment that is the normal maturity of an 11-year-old girl. T2 (Tanner 2) = the first signs of maturation; M = onset of menarche; Risser stage 1 appears after the onset of menarche and between 15 and 16 years a normal girl has fully matured (Risser 5 or Tanner 5). The three coloured lines describe the normal variation of the normal growth velocity (modified according to Weiss et al. 2021).

In this patient, progression of the severe and stiff curve was prevented during the most vulnerable phase of the pubertal growth spurt whilst the clinical result has obviously been improved (Figure 5). Therefore, it can be assumed that the patient may have a normal life with minor restrictions only in adulthood.

There appears to be little specific literature on scoliosis and CHD (Tsirikos et al. 2020). Whilst the older literature is devoted to pathogenesis, the more recent literature is more concerned with outcomes and complications of spinal surgery. Accordingly, we can state that the expressions of scoliosis appearing after heart surgery in early childhood seem variable. Sometimes, scoliosis is not a consequence of such interventions, and some spinal deformities may also be mild. The case as presented here may be considered as being very severe and stiff. Therefore, the concept of a tethered spine because of an internal scar after thoracotomy is a reasonable cause for this condition with a high risk for progression during growth.

According to Ruiz-Iban et al. (2005), thoracotomy causes scoliosis in patients with CHD. According to our hypothesis, the most likely reason for the development of such a strong and stiff curvature in some patients after surgery for a CHD is the development of a scar within the chest cavity in the area of surgical access that does not grow with the patient. However, the concept of such a scar tethered spine has not yet been evaluated. We would suggest evaluating magnetic resonance imaging (MRI) scans of operated patients with CHD with respect to soft tissue strings even if these appear to be subtle.

### Limitations of this article

This is a preliminary report as the patient is not yet fully grown. Considering the facts that (1) most of the progression (two-thirds) occurs whilst the patient is on the ascendent phase of the pubertal growth spurt (Goldberg et al. 2002) and now the patient is in the descendent phase and (2) that the preliminary results at follow-up of at least 18 months are similar to the end results (Weiss et al. 2021), this case already provides valuable information. Now that the patient is maturing and the possible corrective effects reduce (Ishihara & Shiraishi 2021), we can estimate that there will potentially be a loss of the correction achieved intermediately.

A case report *per se* is of limited evidence. However, if there is no existing evidence that describes the outcome of an intervention, the publication of a case report is justified. This case report reveals that high-quality conservative treatment may lead to an improvement of the deformity as a result of a stiff spinal deformity following two consecutive surgeries for CHD in early childhood and may decelerate the curve progression. A systematic review of the literature may reveal additional citations on the topic of conservative treatment of patients with severe scoliosis after surgery for CHD.

## Conclusion

Surgery for CHD in rare cases may lead to stiff spinal deformity. Supported by pattern-specific high correction exercises and braces, these typical single thoracic curves can be re-compensated to a more balanced appearance, and thus be less prone to progression in adulthood. Because of the relative high risks of spinal fusion and the long-term unknowns of such an intervention, high-impact conservative treatment should be implemented first before surgical correction is considered.
